# High-performance solid state supercapacitors assembling graphene interconnected networks in porous silicon electrode by electrochemical methods using 2,6-dihydroxynaphthalen

**DOI:** 10.1038/s41598-018-28049-x

**Published:** 2018-06-25

**Authors:** Cosmin Romanitan, Pericle Varasteanu, Iuliana Mihalache, Daniela Culita, Simona Somacescu, Razvan Pascu, Eugenia Tanasa, Sandra A. V. Eremia, Adina Boldeiu, Monica Simion, Antonio Radoi, Mihaela Kusko

**Affiliations:** 1 National Institute for Research and Development in Microtechnology (IMT-Bucharest), 126A Erou Iancu Nicolae Street, 077190 Voluntari, Romania; 2 ‘Ilie Murgulescu’ Institute of Physical Chemistry, Romanian Academy, 202, Splaiul Independentei, Bucharest, 060021 Romania; 3 Faculty of Applied Sciences, Politehnica University of Bucharest, 313 Splaiul Independentei, Bucharest, 060042 Romania; 4 Faculty of Physics, University of Bucharest, 405 Atomistilor Street, 077125 Magurele, Romania; 5 Centre of Bioanalysis, National Institute of Research and Development for Biological Sciences - Bucharest, 296 Splaiul Independentei, Bucharest, 060031 Romania

## Abstract

The challenge for conformal modification of the ultra-high internal surface of nanoporous silicon was tackled by electrochemical polymerisation of 2,6-dihydroxynaphthalene using cyclic voltammetry or potentiometry and, notably, after the thermal treatment (800 °C, N_2_, 4 h) an assembly of interconnected networks of graphene strongly adhering to nanoporous silicon matrix resulted. Herein we demonstrate the achievement of an easy scalable technology for solid state supercapacitors on silicon, with excellent electrochemical properties. Accordingly, our symmetric supercapacitors (SSC) showed remarkable performance characteristics, comparable to many of the best high-power and/or high-energy carbon-based supercapacitors, their figures of merit matching under battery-like supercapacitor behaviour. Furthermore, the devices displayed high specific capacity values along with enhanced capacity retention even at ultra-high rates for voltage sweep, 5 V/s, or discharge current density, 100 A/g, respectively. The cycling stability tests performed at relatively high discharge current density of 10 A/g indicated good capacity retention, with a superior performance demonstrated for the electrodes obtained under cyclic voltammetry approach, which may be ascribed on the one hand to a better coverage of the porous silicon substrate and, on the other hand, to an improved resilience of the hybrid electrode to pore clogging.

## Introduction

Surface reactions involving adsorption/desorption of ions and transfer of electrons at the electrode/electrolyte interface may give rise to charge accumulation, thus promoting the development of electrochemical devices known as electric double-layer capacitors (EDLCs) or pseudocapacitors (PCs), respectively. The ability of EDLCs and PCs to perform as energy storage systems (ESSs) is strongly related to the electrode material(s), electrolyte features (aqueous or organic, conductivity, ionic size, etc.)^1^, separator and current collector.

The large scale use of carbon, as activated carbon in most commercially available EDLCs^2^, is ascribed to its fair conductivity, high surface area (500–3000 m^2^/g)^3^, and relatively low cost procurement. Carbon based materials (carbon nanotubes^4^, carbon onions^5^, carbide derived carbons^6^, mesoporous carbons^7^ or carbon aerogel^8^ have been thoroughly investigated and reported^9–11^ as variant forms competing against activated carbon. Graphene has a special place in the family of carbonaceous materials due to its peculiar properties^12^, and because of such reason, efforts have been made for its functionalization in order to be used as high performance active electrode material, aiming to also control efficiently the interlayer distance between the graphene sheets in order to increase the accessible surface area for the ionic liquid electrolyte^13^. Recently, mesoporous graphene electrodes showed capacity of achieving simultaneously high power and energy densities^14^. Synergistic outcomes were reported for and Ni-Co hydroxide nanoneedles embedded in graphene hydrogel providing fast ionic transport, binder free and high redox active electrode material^15^. Furthermore, mainly for developing flexible devices, either carbon or graphene derivatives were used in fabrication of various hybrid organic electrode materials, such as polyimide-coated carbon electrodes^16^, carbon-redox-polymer-gel electrodes^17^, graphene-polypyrrole composite^18^ or pyrene-functionalized poly(2,2,6,6,-tetramethylpiperidinyl-1-oxyl methacrylate)/rGO layered composites^19^.

Silicon, which is the second most abundant element on our planet, is also used in the supercapacitor applications, especially in its nanostructured forms, silicon nanowires or porous silicon. Understanding the behaviour of ions in (nano)pores is still under debate^2^, their mobility being influenced by the pore size and shape, and since not all the pores grant access to ions, there is no linear relation between the capacitance and the specific surface area. Accordingly, the first attempts were focused on finding the suitable geometry for nanostructured silicon, and bare, unmodified porous silicon (p-Si)^20^, vertically aligned^21^ or randomly deposited silicon nanowires^22^, as well as branched silicon nanowires^23^, but, when used as electrodes, the resulted supercapacitors showed modest capacitance between 0.2 and 0.9 mF/cm^2^ (equivalent to 5–20 mF/g). On the contrary, when porous silicon, as ultra-high surface area material (up to 800 m^2^/g), provides the frame for different complementing materials^24–26^ the capacitance values rises, and capacitance densities as high as 325 mF/cm^2^ were achieved when a thin carbon layer was deposited on porous silicon nanowires^27^. If initially raised as scaffold material for carbon derivatives or conducting polymers^28^, recently more elaborated techniques are proposed for the modification of the Si nanostructures’ surface by coating with thin layers of active materials^29,30^ or decorating with nanoparticles^31,32^ to further enhance the supercapacitor performance, and the pseudocapacitive materials were seriously considered in these attempts.

In this context, the molecules bearing quinone moiety are promising candidates for enhancing electrochemical devices due to their redox ability via quinone-hydroquinone couple. Therefore, quinone-based materials have attracted considerable attention as electrode constituents for ESSs, such as lithium-ion batteries^33^, redox flow batteries^34^, polymer/air batteries^35^ and electrochemical capacitors^36–38^. The ability of 1,4-dihydroxynaphthalene derivatives as organic additives for activated carbon electrodes of supercapacitors has been recently reported^39^, the specific capacitance enhancement being ascribed to the formation of pseudocapacitance by the quinone-hydroquinone couple in the composite electrode. This ability was also explored as redox-active additives in either simple H_2_SO_4_ or polyvinyl alcohol (PVA) - H_2_SO_4_^40^ or gel polymer supporting electrolytes^41^ enabling further fabrication of dual redox system based on different quinones^42^.

When the fabrication of solid state supercapacitors on silicon is planned, an important drawback that has to be overcome is finding the pathway to assure the uniformity, stability and the completeness of internal surface modification. Electrochemical grafting of acrylate monomers^43^ allowed widening the operating potential window of a supercapacitor through an electrically-insulating polymeric layer at the negative carbon electrode. 2-aminoanthraquinone electrochemical immobilization performed at a composite electrode made of activated carbon, acetylene black carbon, graphite and polytetrafluoroethylene (as binder) increased the stability of the grafted molecule, therefore improving the features of the supercapacitor^44^.

Herein, we propose the modification of porous silicon by electrochemical deposition of 2,6-dihydroxynaphthalene (2,6-DHN), either by cyclic voltammetry or potentiometry, resulting after thermal treatment (800 °C, under N_2_) an assembly of interconnected graphene networks in porous silicon electrode. Nonetheless, the electro-deposition process is not only an accessible, cost effective and scalable method, but, more important, it allows to conformally incorporate the filler materials, even when nanoporous template is used^45^. The charge storage features of the symmetric solid state supercapacitors (SSC) built using as electrolyte PVA-H_2_SO_4_ gel were investigated and remarkable performances were obtained, governed by the manner of assembling the nano-carbon material inside of porous structure and consequently by the micro-structural characteristics. Accordingly, both SSCs exhibit a high energy density and an ultra-high power density when operated at 2.1 V; thus, *NC_ J*-*SSC* displays the highest specific energy of 24.8 Wh/kg (corresponding power density of 420 W/kg), while *NC_CV-SSC* is capable of delivering the highest power density 53.8 kW/kg at an energy density of 7.6 Wh/kg. Both devices offer a good capacity retention, close to 80%, at a high current density of 10 A/g after 1000 cycles, with a favourable behaviour for *NC_CV-SSC*.

## Experimental

### Fabrication of the porous silicon (p-Si) and its modification

The porosification process of Si wafers (B doped, 1–5 mΩ∙cm resistivity, (100) crystallographic orientation, provided by SIEGERT WAFER GmbH) was achieved through electrochemical etching, anodically polarizing the wafers at constant current density of 10 mA/cm^2^ during 300 s, in 1:1 (v/v) electrolyte solution (40 wt. % HF and 98 wt. % ethanol) using a single cell bath for porous silicon formation on 4 inch wafers (AMMT GmBH, Germany). Previously, the wafers have been cleaned using the Piranha solution (97 wt. % H_2_SO_4_ and 30 wt. % H_2_O_2_, 3:1 v/v), at 80 °C, for 30 minutes and finally rinsed with deionized water and dried under N_2_. Native oxide layer was removed during few-minutes dipping in 5% (v/v) HF. At the end of the anodization process, the porosified wafers were washed with ethanol and kept during 30 minutes in isopropanol in order to minimize the mechanical stress, thereafter being blown under nitrogen and retained for further use. Porous silicon (p-Si) pieces (1.2 × 1.8 cm) were dipped for 5 minutes in 5% (v/v) aqueous HF, plenty rinsed with deionized water and dried under N_2_. An electrochemical cell was assembled using porous silicon as working electrode (WE), a saturated calomel electrode as reference electrode (RE) and a Pt wire as counter electrode (CE), the electrolyte, consisting of 10 mM phosphate buffer saline solution, pH 7.40, containing 0.1 M KCl. Electrodeposition was achieved either by cyclic voltammetry (5 mV/s, potential window between −0.45 V and +0.45 V, during 6 cycles) – samples noted *NC_CV* – or potentiometry (1 mA, 120 s) – samples noted *NC_ J* – using a 2 mM solution of 2,6-dihydroxynaphthalene (2,6-DHN) dissolved in the electrolyte solution. The electrodeposited p-Si pieces were cleaned with deionized water, dried under N_2_ and thermally treated at 800 °C, during 4 hours, under N_2_ flow (Nabertherm tube furnace, model R 50/250/12).

### Symmetric supercapacitor device assembly

A Meltonix gasket membrane (60 µm thickness, 7 × 7 mm^2^ exposed area) was used as separator and few drops of gel electrolyte (6 g of polyvinyl alcohol - average Mw 85 000–124 000, 87–89% hydrolysed - dissolved in 60 mL of hot water (85 °C) containing 6 g of H_2_SO_4_) fused the two p-Si modified pieces. Following a similar process flow, for each of the two types of modified p-Si electrodes symmetric supercapacitor devices were fabricated, *NC_CV-SSC* and *NC_ J-SSC*, respectively.

## Characterization

### Materials characterization

The samples were morphologically characterized by using a field emission scanning electron microscope (FE-SEM), FEI-NOVA NanoSEM 630, nitrogen adsorption/desorption isotherms at 77 K recorded on a Micromeritics ASAP 2020 analyzer and different X-ray diffraction techniques recorded with Rigaku SmartLab diffraction system, with CuK_α1_ wavelength (1.5405 Å) in parallel beam (PB) mode. The samples were degassed at 523 K for 2 hours under vacuum before nitrogen adsorption analysis. Specific surface areas (S_BET_) were calculated according to the Brunauer-Emmett-Teller equation using adsorption data in the relative pressure range 0.05–0.3, while pore size distributions were obtained from the desorption branch using the Barrett–Joyner–Halenda (BJH) model. The total pore volume (V_total_) was estimated from the amount adsorbed at the relative pressure of 0.99.

Surface analysis performed by X-ray photoelectron spectroscopy (XPS) was carried out on PHI Quantera equipment with a base pressure in the analysis chamber of 10^−9^ Torr. The X-ray source was monochromatized Al K_α_ radiation (1486.6 eV) and the overall energy resolution is estimated at 0.65 eV by the full width at half-maximum (FWHM) of the Au4f7/2 photoelectron line (84 eV). Although the charging effect was minimized by using a dual beam (electrons and Ar ions) as neutralizer, the spectra were calibrated by using the C1s line (BE = 284.8 eV).

The mass of active electrode materials was estimated using a microbalance (XS 205 Mettler-Toledo, 0.01 mg precision) by weighing samples of unmodified silicon, as-prepared porous silicon, *NC_ J* and *NC_CV* electrodes. Following the procedure described in the section *Estimation of active electrode mass* from Supplementary Information document, the active mass of *NC_ J* electrode was calculated to be 90.8 µg while the *NC_CV* was found to be 74.2 µg.

### Electrochemical tests of symmetric solid state supercapacitors

The obtained assembly was tested using an Autolab 302 N equipped with FRA 32 M, SCAN 250 and ADC 10 M modules. Galvanostatic charge-discharge (GCD) measurements were performed using currents ranging between 5 µA–1 mA, voltammograms (CV) were acquired in the range 5 mV/s–5 V/s and electrochemical impedance spectra (EIS) were recorded in the 0.1 MHz–10 mHz interval, at 0 V DC and 10 mV AC bias.

## Results and Discussion

### Morpho-structural and chemical characterization of the nanocomposite p-Si based electrodes

**Figure 1:**
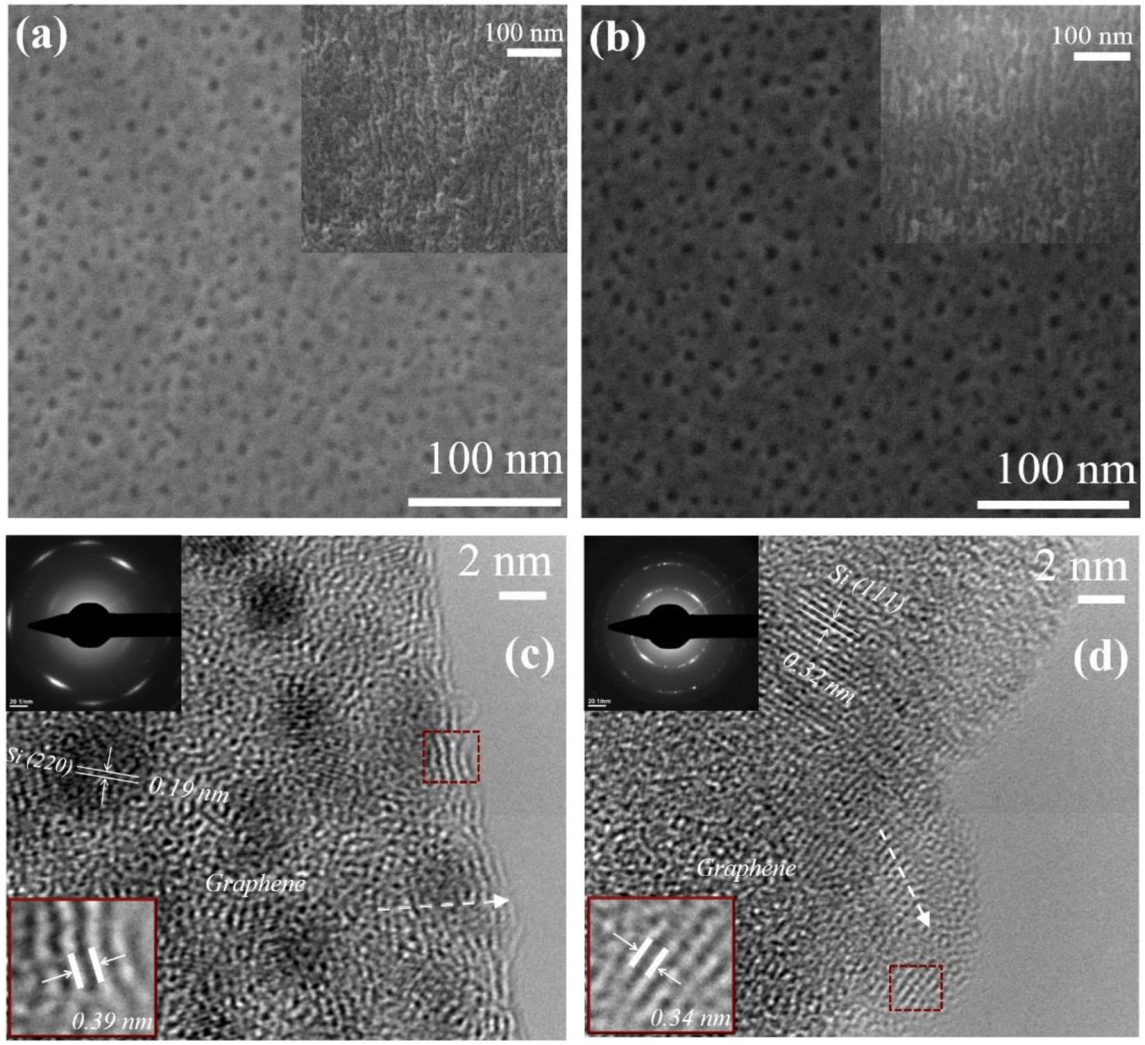
HR-SEM plan view images of modified porous silicon layer, corresponding to *NC_ J* (**a**) and *NC_CV* (**b**) electrodes, respectively (cross-section detail – inset image); HR-TEM images of micropores for structure *NC_ J* (**c**) and structure *NC_CV* (**d**), respectively; upper inset: the corresponding SAED pattern.

The Fig. 1 displays the acquired micrographs during the morphological investigations of the modified p-Si electrodes.

**Figure 2:**
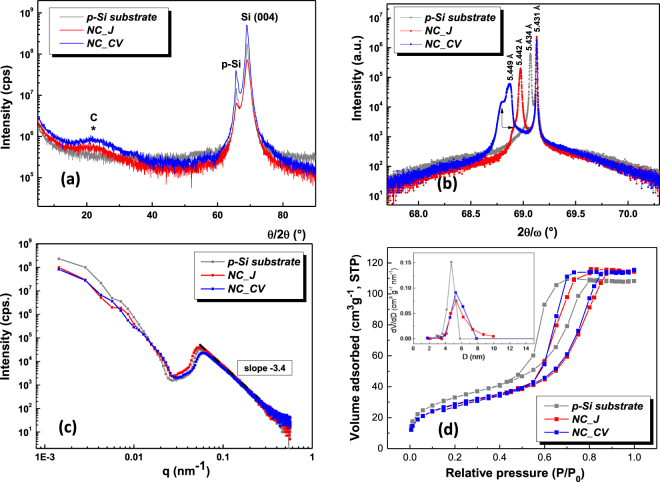
X-ray diffraction analyses: (**a**) *θ/2θ* wide angle X-ray diffraction; (**b**) *2θ/ω* scans for (004) Bragg reflections; (**c**) small angle X-ray scattering; and (**d**) N_2_ adsorption/desorption isotherms for *NC_CV* and *NC_ J* samples in comparison with *p-Si substrate*.

There are no significant morphological changes between the p-Si starting substrate and the carbonized samples and this evidence suggests that during the electrochemical polymerization step an ultra-thin film coated the pore walls^46^. The top-view HR-SEM images (Fig. 1(a,b)) demonstrate that the pores remained open and unblocked, showing a uniform distribution of pores, with diameters ranging between 5 to 12 nm, with an average around 6 nm. The cross-sectional SEM images (inset in Fig. 1(a,b)) demonstrate the branch like morphology of electrodes with nanochannels which provides ionic pathways for effective diffusion of electrolyte, slightly larger when the polymer was deposited by cycling the voltages prior carbonization. A closer inspection of the resulted hybrid electrodes was further realized performing HR-TEM analyses and the results are shown in Fig. 1(c,d). The images from sidewalls fragments reveal that a highly interconnected network of randomly orientated graphene layers encase the silicon pores which show up as lattice fringes with interplanar d-spacing of 0.19 nm and 0.32 nm associated with (220) and (111) planes of cubic Si, respectively. The SAED diffraction ring and spot patterns further confirm the presence of localized short range ordering. Graphene layers are either isolated or stacked with the graphite (002) lattice spacing of 0.34 nm or larger (shown in the magnified marked area) owing to the globally disordered feature of the network^47^. The crystal structure and phase of hybrid graphene/p-Si were systematically examined by X-ray diffraction measurements (Fig. 2(a–c)).

Firstly, wide angle X-ray diffraction (*θ/2θ*) was performed to confirm the existence of nano-carbon inside of porous silicon – Fig. 2(a). In comparison with *p-Si substrate*, both XRD patterns of the *NC_CV* and *NC_ J* samples present clearly defined Si assigned peaks, including the supplementary ones arising due to nanostructuration process (noted p-Si), but also the peaks specific for carbon based materials. These broad and smaller peaks located at ~22.1° (marked as *C in graph) indicate a low degree of graphitization of nanocarbon ultrathin film^48^. The 2θ value is very close to those typically found for graphene quantum dots (GQDs) and indicates an increase of the lattice parameter against graphite, resulted from the adding of disordered carbon belonging to non-graphitized polymer chains^49^. The interplanar distances and the mean crystallite size were calculated using $\lambda \,=\,2d\,{\sin }\,\theta$ Bragg’s law (where *d* is the interplanar distance, *λ* is the wavelength of the incident X-ray, *θ* is the Bragg’s angle). Accordingly, the carbon related peak corresponds to a lattice parameter of 4.02 Å, which was assigned either to the (002) lattice spacing graphene sheets in functionalized GQDs^50^ or incipient double wall carbon nanotubes^51^. For an in-depth analysis of Si lattice, 4 - bounce Ge (220) monochromator at incidence that provide an angular precision of 0.003° were used for high resolution measurements – Fig. 2(b). Studying the specular scattering, we observe that the value of bulk Si lattice was preserved, while the lattice corresponding to porous silicon was modified after the partial carbonization, from 5.434 to 5.442 and 5.449 Å, respectively, the largest shift being obtained for *NC_CV* sample. Besides an increasing of p-Si lattice constant, an additional stress of p-Si (left shoulders – arrow marked) was revealed^52^. Analysing the diffuse scattering, mean pore radius was estimated for both samples, around 6 nm, in excellent agreement with SEM results. Furthermore, the *NC_CV* sample presents a higher integral area of the p-Si related peak and also a higher lattice value, which indicated an increased porosity. In order to study the nanocomposite material density, X-ray reflectivity (XRR) measurements were performed – Fig. S2. The values of the critical angle increased from 0.29° from *p-Si substrate* to 0.30° (*NC_CV*) to 0.36° (*NC_ J*), which demonstrate that the deposition mostly occured in the depth of porous matrix, covering the Si nanofibrils, and the samples’ surface generally remained unaffected.

Unlike XRD, small angle X-ray scattering (SAXS) measurements are sensitive to fractal surface of p-Si and are able to estimate specific surface area. Two linear parts separated by a curved region can be identified in the SAXS profiles shown in Fig. 2(c), where the values of scattering vector *q* $(q=\frac{4\pi }{\lambda }\,{\sin }\,\theta )$ ranged from 0.002 to 0.570 Å^−1^. The curved region, which is related with the volume of voids in structure, is clearly changed for *NC_ J* and *NC_CV* samples against the reference p-Si, consistent with the deposition of ultra-thin film of polymer on the pores’ walls. On the contrary, there are no remarkable differences between the p-Si samples prior and after carbonization for the linear region located at larger scattering vectors, which corresponds to the region of interfaces, and moderate differences appear in the second region, located at small *q*, which is related to the bulk of samples^53,54^. Consequently, whereas the p-Si surface did not change significantly after polymer deposition/carbonization, the pores’ morphology was altered. At large q-values, the intensity followed a Porod’s law decay with *I(q) ~q*^*−α*^. Fitting the SAXS profiles, a non-integer value for *α* was obtained, *α* = *−3.4*, which indicated that the active material of the electrode is a porous system having a fractal nature of its structural morphology^55^. From a qualitative point of view, since scattering intensity are similar, *NC_ J* and *NC_CV* have a comparable specific surface area, smaller than the pristine reference p-Si sample. Quantitatively, specific surface areas of the investigated materials (*S*_*n*_) were calculated using the following formulas:

1 $${S}_{n}=\frac{\pi p(1-p)}{\rho }\frac{{K}_{p}}{Q}$$

2 $$\mathrm{ln}\,\{{q}^{3}I(q)\}=ln{K}_{p}+{\sigma }^{2}q$$

3 $$Q={\int }_{0}^{\infty }qI(q)dq$$

where *p* and *ρ* are the sample porosity and density, respectively, *K*_*p*_ is the Porod constant calculated with formula (2) from the asymptotic behaviour of the tails in the high *q* region, and *Q* is Porod invariant given by the Porod integral (3), *I(q)* scattering intensity, and *σ* is a parameter characterizing the state of the interface surface.

The parameters obtained using the above formalism are presented in Table 1. It is notable that the internal surface area resulted for *NC_CV* sample is 141.46 m^2^g^−1^, larger than the value obtained for *NC_ J* sample, i.e. 125.01 m^2^g^−1^. Both values are smaller than the one obtained for the reference sample, confirming that the pores were diminished due to the conformal coverage of the Si fibrils with carbonic material. Moreover, the slight increase of the mean pore radius might be related to the collapse or dissolution of thinnest fibrils during the electrodeposition process.

**Table 1:** Textural parameters of *NC_CV*, *NC_ J* and *p-Si substrate*.

Sample	k_p_ (Å^−2^)	Q (Å^−3^)	S_SAXS_ (m^2^g^−1^)	r_XRD_ (nm)	S_BET_ (m^2^g^−1^)	S_micro_^*^ (m^2^g^−1^)	V_total_ (cm^3^g^−1^)	r_BET_ (nm)
*NC_CV*	6.15	473	141.46	6.0	100.9	4.1	0.178	5.4
*NC_ J*	6.09	530	125.01	6.1	99.3	4.8	0.177	5.7
*p-Si substrate*	1.01	55	199.79	5.8	115.5	6.4	0.167	4.6

^*^The micropore surface area was determined via t-plot analysis.

The porous structure of the obtained hybrid electrodes was also verified by N_2_ adsorption/desorption measurements and the corresponding isotherms are shown in Fig. 2(d). Both isotherms are type IV according to the IUPAC classification^56^. The H_2_ type hysteresis loops which closes around P/P_0_ = 0.5 in both samples indicates the presence of an interconnected pore system in which mesopores are predominant (~95–96%). The small fraction of micropores of around 4–5% determined for both samples and also for the *p-Si* substrate was probably caused by the electrochemical etching of Si wafers. The average pore diameter calculated using the desorption branch is almost similar for both samples (inset graph), 5.4 nm for *NC_CV* and 5.7 nm for *NC_ J*, slightly larger than *p-Si* substrate (4.6 nm). Both values are in good agreement with the average pore sizes derived from SEM and XRD measurements. The decrease of specific surface areas (S_BET_) at the same time with the increase of average pore diameters for both samples as compared to the *p-Si substrate* was predictable after electrodeposition due to partial obstruction of the pores. As shown in Table 1, S_BET_ and total pore volume values (V_total_) resulted are almost similar for the two samples, but nevertheless, there are notable discrepancies between the values obtained using this method in comparison with previous SAXS analyses in terms of surface area. If N_2_ adsorption measurements led to a surface of around 100 m^2^g^−1^ for both samples, slightly larger for *NC_CV*, the SAXS measurements revealed higher values, but they are reasonable, since the X-ray analyses are able to see the total internal surface (both open and closed porosity), demonstrating that a significant part of the pores from the nanocomposite network are inaccessible for the N_2_ gas^57^. The existence of ultramicropores (in the Angstrom range) within the graphene-porous silicon interconnected network should be favourable for enhancing the ionic transport behaviour^58^, especially when it is used an electrolyte containing small ions, like H^+^, and thus improve the electrochemical performance as supercapacitor electrodes.

The structural parameters obtained using SAXS, XRD and N_2_ adsorption/desorption measurements are summarized in Table 1.

The XPS method was used to investigate the surface chemistry (<10 nm) of the modified p-Si substrates. The high resolution C1s spectra clearly identify its chemical bonding and, after data quantification, the percentages of the associated chemical species (Figs S2, S3 and Table S1). It is appropriate to notice that our experimental errors for the binding energies (BEs) assignments are within ± 0.2 eV, while for the quantitative analysis (relative concentrations) in the range of ±5%. Figure S2(a,b) shows the deconvoluted C1s spectra exhibiting the typical features of the graphene according to Voiry assignments^59^. Thus, the sp^2^/sp^3^ hybridised carbon were accommodated under the symmetric shape of the C 1s spectra, while the oxygen functional groups (C–O, C=O, O=C–O) were fitted under the asymmetric side with the characteristic binding energies (BEs) shown in Table S1. The data reveal differences in the behaviour of the samples *NC_CV* and *NC_ J*. Thus, the *NC_ J* sample shows a higher percentage of oxygen functionalities (21.3%, overall) as compared to the sample *NC_CV* (16.0%, overall). The most pronounced relative concentration is associated to C-O chemical species (16.0% for the sample *NC_ J* in comparison with 10.9% pertaining to the sample *NC_CV*). The C – sp^2^ (graphitic) hybridization percentage display a larger value in the *NC_ J* sample (see Table S1).

The largest percentage of oxygen is bonded to the silicon substrate as SiO_2_ (see Fig. S3(a)). A small amount of oxygen is bonded to the carbon leading to the aforementioned oxygen functionalities. All these chemical species are accommodated under the same envelope. Regarding the substrate chemistry, Si 2p XPS spectra clearly display elemental Si (Si°) and full oxidized Si (Si^4+^) as the substrate materials (Fig. S3(b)). A close inspection of the spectra as well as the numerical values (Table S1) indicate differences between our samples. Thus, Si° amounts for ~ 65.8% in the *NC_ J*, while the same species is lower (54.8%) in the sample *NC_CV*.

### Electrochemical performances of the symmetric supercapacitors assembled using modified of p-Si electrodes. Comparative analysis of *NC_ J-SSC* versus *NC_CV-SSC*

**Figure 3:**
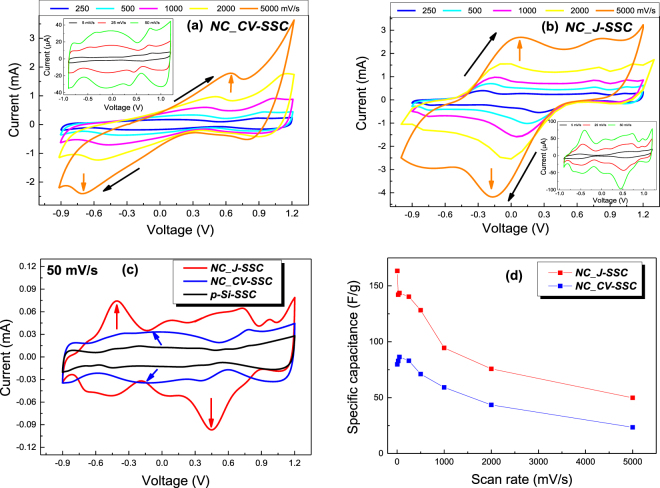
Cyclic voltammograms at different scan rates ranging from 5 to 5000 mV/s for *NC_CV-SSC* (**a**) and *NC_ J-SSC* (**b**) devices; (**c**) Comparative CV curves obtained at scan rates of 50 mV/s with respect to reference *p-Si-SSC*; (**d**) Variation of SSC specific capacitance with the scan rate.

Symmetric quasi-solid state supercapacitors (SSC) were assembled using modified of p-Si electrodes and standard PVA/H_2_SO_4_ gel electrolyte and their electrochemical performances were firstly investigated using standard cyclic voltammetry (CV) and galvanostatic charge discharge (GCD). Figure 3 shows CVs of the fabricated devices, both *NC_CV-SSC* and *NC_ J-SSC*, within potential window from −0.9 V to 1.2 V, recorded at scan rates ranging over three orders of magnitude between 5 mV/s to 5 V/s.

Comparing with the reference *p-Si-SSC*, the increase of the CV curves area is clearly visible for both capacitors resulted using the modified electrodes – Fig. 3(c). As it can be observed, while the previously reported p-Si based SSCs, both pristine and graphene coated^24^, exhibit CV curves with rectangular shapes, in our case, both devices do not show an ideal capacitive response, indicating the significant contribution of pseudocapacitance. It is even more accentuated for *NC_ J-SSC*, where an additional, intense couple of redox peaks is also observed, which causes higher current density and largely enhances the CV area. There are reports showing that faradaic reactions could be promoted via utilisation of the PVA/H_2_SO_4_ gel electrolyte^60^, which is feasible since small features can be also observed in the voltammogram recorded for *p-Si-SSC*. However, in our case they are augmented by the presence of oxygen functionalities and the defective sp^3^ sites in graphene shell arising from 2,6-DHN polymer partial carbonization. The prominent peaks (arrow marked in Fig. 3(a–c) are the solely ascribed to a diffusive behaviour and are visible even at extremely high scan rate up to 5 V/s. The potential window encompassing these peaks is very large, spanning almost for 1 V, for *NC_ J-SSC* the diffusive peaks being located at the limits of the extended potential window differentiating from *NC_CV-SSC* where very large and poorly defined peaks manifest. In the latter case the broadened peak indicates that the capacitance mainly originates from the electric double layer capacitance, minor pseudocapacitance contribution being ascribed to oxygen functionalities. The nature of the redox reactions involved at *NC_ J-SSC* and *NC_CV-SSC* may be fairly ascribed to quinone-hydroquinone couple (Q-QH_2_)^61^, the decomposition of quinones occurring above 800 °C^62^. Hydroquinone is known to possess a rather reluctant transfer of electrons, its behaviour at Pt electrodes being ascribed to an irreversible electrochemical^63^ transfer of electrons as in our case where the overpotential of the Q-QH_2_ couple at the p-Si electrode is drastically increased, lowering the charge transfer rate of the Q-QH_2_ redox reaction. *NC_ J* sample is richer in oxygen functionalities (C-O/C-OH, C=O/O-C-O and O=C-O) whereas *NC_CV* bears more carbon (+40%), thus *NC_ J-SSC* is highly oriented towards pseudocapacitance performance meanwhile *NC_CV-SSC* should promote better cycling stability features compared to *NC_ J-SSC*. Furthermore, incorporating oxygen makes the surfaces more hydrophilic, and consequently more wettable towards aqueous based electrolytes. In our case ultrathin layers made of interconnected network of graphene sheets with locally-ordered domains preserved the chemical instable porous silicon surface, allowing the silicon carbon passivated interface to be accessed by solvated ions and interrogated by redox active species. Barrier made of few-layered graphene or thin carbon materials are suitable against corrosion and remain electrically conductive^26^. The CV curves present in both cases a symmetric increment of specific current with scan rate that might represent a preliminary sign of outstanding supercapacitive behaviour of the SSC devices. In this context, it is remarkable that when compared to conventional electrodes prepared from carbon materials, even for mesoporous or hierarchical carbon, use of p-Si as support and, furthermore, employment of an electrochemical process for internal pore surface carbonization, we were able to use ultrahigh rates for voltage sweep, of 5 V/s. Similar performances were reported for electrodes obtained based on silica-templated ordered mesoporous carbon thin films, but the potential window in that case was substantially smaller, only between 0 and 1 V^64^. Regarding the operational voltage range, a 2.1 V voltage window was obtained, slightly lower than the ones reported for SSC based on graphene-coated p-Si, but filled with 1-ethyl-3-methylimidazolium tetrafluoroborate (EMIBF_4_) electrolyte (e.i. 2.3 V)^24^ or tetraethylammonium tetrafluoroborate (TEATFB) electrolyte (i.e. 2.6 V)^26^.

Based on CV measurements the specific capacitances of the devices were calculated according to the following equation:

4 $$C=\frac{1}{\upsilon ({V}_{f}-{V}_{i})m}{\int }_{{V}_{i}}^{{V}_{f}}I(V)dV$$

where *υ* is the scan rate (V/s), *V*_*i*_ and *V*_*f*_ are the limits of the sweep potential window, *m* is the mass of the active material and *I(V)* is the voltage-dependent current response.

The specific capacitances of the supercapacitors *NC_ J-SSC* and *NC_CV-SSC* at a scan rate of 5 mV/s were determined to be 163.4 and 79.8 F/g, respectively, values that place our devices among the best reported performances. As it can be observed in Fig. 3(d), the specific capacitance gradually decreases with the increase of scan rates, slightly more accentuated in the case of *NC_ J-SSC*. This behaviour is a natural consequence of time constraint, when the diffusion of protons is limited at high scan rates. Nevertheless, it is remarkable that the values remain considerable higher even at large sweep rates, about 100 and 60 F/g at 1 V/s, the last measured values being around 50 and 24 F/g at 5 V/s. Though the electrochemical tests over such a large range of sweep rates are only scarcely reported, a substantially improved rate capability is achieved compared to the loss recorded from 113.5 F/g at 1 mV/s to approximately 20 F/g at 1 V/s when graphene/MnO_2_ nanocomposite was used as electrode material^65^. The superior rate capability obtained can be attributed to the reduced short diffusion path of ions, but, more vital in our new systems, might be the excellent interfacial contact between the graphene-like interconnected networks and the highly doped porous silicon electrode that provide electronic conductive channels allowing the fast transfer of electrons throughout the whole electrode matrix.

**Figure 4:**
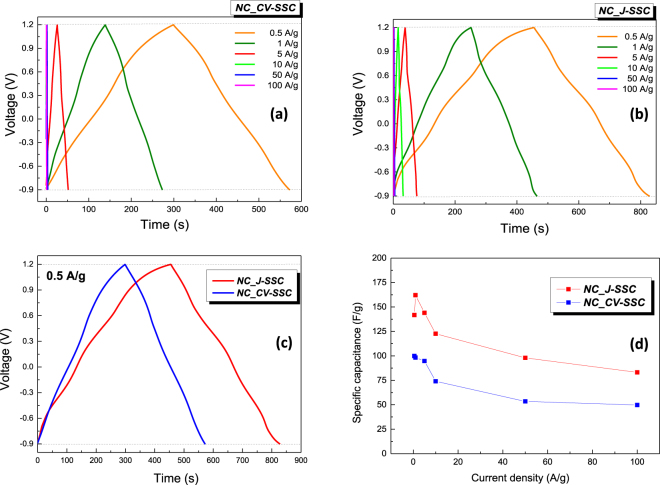
Charge discharge curves measured over the potential window of −0.9–1.2 V at different current densities ranging from 0.5 to 100 A/g for *NC_CV-SSC* (**a**) and *NC_ J-SSC* (**b**) devices; (**c**) Comparative of the charge-discharge profiles measured at a constant current density of 0.5 A/g; (**d**) Variation of the specific capacitance with the current densities.

The charge discharge profiles at constant current densities ranging from 0.5 A/g up to ultra-high 100 A/g are depicted in Fig. 4(a,b) and revealed different regions: firstly an initial very small IR ohmic drop due to internal resistance and interchange of linear sections and curve regions corresponding to the concurrent mechanisms of double layer and faradaic charge storage. The associated ohmic drop values for different current densities are plotted in Fig. S4, both supercapacitors having reduced internal resistance, allowing high discharge power delivery. Charge-discharge curves indicated for NC_ J-SSC the longest discharge time (36% greater than value for NC_CV-SSC) (Fig. 4(c)) which is consistent with the CV recordings.

Taking into account that both devices are symmetrical systems, working as non-ideal EDLCs, the specific capacitance *C* is deduced from the discharge curve using the relationship^66^:

5 $$C=\frac{2I\int Vdt}{m\,{({V}_{f}-{V}_{i})}^{2}}$$

where *I/m* is the discharge current density (A/g), *V*_*i*_ and *V*_*f*_ are the initial and final values of potential operational window and ∫*Vdt* is the integral current area.

As it can be observed, both devices exhibit very high charge storage capacity, with a significant specific capacitance even when the current density reaches the ultrahigh value of 100 A/g (200 times of initial current density) – Fig. 4(d). In the case of *NC_CV-SSC* the highest delivered specific capacitance was 100 F/g at a current density of 0.5 A/g, and it still retained 50 F/g at 100 A/g, decreasing only at half of its initial value. The current response of the second capacitor improves additionally the previous one’ figure of merit, i.e. the highest specific capacitance is 162.4 F/g at the current density of 1 A/g and losses only 51% from its initial value at 100 A/g, although the presence of pseudo-capacitance phenomena, characterized by a slower kinetics than double-layer formation, is more accentuated in this case. The excellent rate capability validated by GCD results confirms the one achieved by the stable voltammograms recorded over wide range of sweep rates and demonstrates that the large ion storage capacity is accompanied with an enhanced ion transport capability.

**Figure 5:**
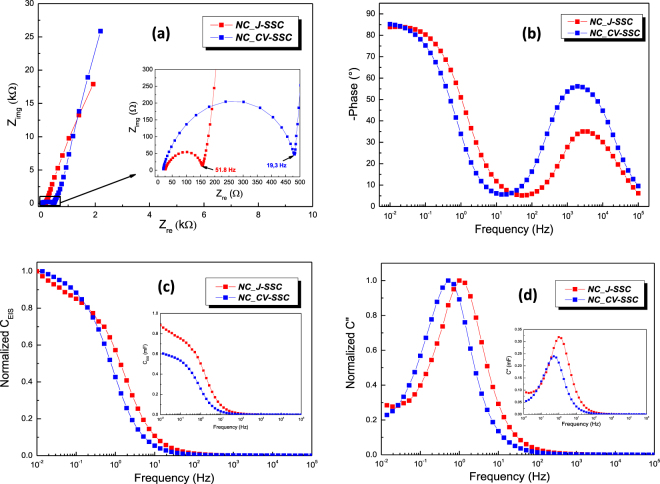
Electrochemical impedance spectroscopy measurements for *NC_CV-SSC* and *NC_ J-SSC* devices: (**a**) Nyquist plots (inset: high frequency detail plots); (**b**) Bode phase angle plots as function of frequency; (**c**) variation of capacitance (C_EIS_) with frequency – normalized and absolute (inset) values; (**d**) variation of imaginary capacitance (C”) with frequency – normalized and absolute (inset) values.

Towards clarifying the mechanism of charge storage and in-depth understanding of the capacitive nature of electrode material in an electrode/electrolyte interfacial system, electrochemical impedance spectroscopy (EIS) measurements were carried out and data were analyzed using both Nyquist and Bode plots - the comparative graphs are displayed in Fig. 5.

As evident from the Nyquist and Bode plots, Fig. 5(a,b), at high frequency the SSCs act like a resistor, while they show capacitive behaviour when the frequency decreases. The impedance spectra were further analysed using the ZSimpWin software on the basis of the electrical equivalent circuit *R*_*s*_*(Q*_1_*R*_*1*_*)(C*_*2*_*(R*_*2*_*(Q*_*3*_*(R*_*3*_*Q*_*w*_*)))* Fig. S5(a) and the simulated curves are showed comparatively with the experimental ones in Fig. S5(b,c), while the fitted impedance parameters, as well as the corresponding standard deviation of the overall fit (*χ*^2^), are presented in Table S2. The circuit model takes into consideration the dual nature of the charge storage, as well as the high porosity and the nanocomposite microstructure of the electrodes, and resulted mean errors ranging between 1E-5–1E-4 confirm the excellent fitting and the complete assessment of the electrochemical processes occurring on/within the system. Therefore, the circuit contains, besides the solitary RC-parallel network (on the left-hand side of the diagram) that represents the pinhole effect specific for polymer based electrolyte^67^, a simplified vertical transmission line type circuit for the two level of hierarchy in the pore network according to our nanocomposite electrode^68,69^. Moreover, the inhomogeneity of the electrode/electrolyte interface and highly branched structure of the electrode recommend the operation with a constant phase element instead of the storage related capacitances (*Q*_1_, *Q*_3_), as well as a replacement for classic Warburg diffusion element (*Q*_*W*_)^70,71^. *Rs* is calculated from the intersection of the plot on the real axis in the high-frequency region and represents a combined resistance related to twice ohmic resistance of the individual electrode (intrinsic resistance of the substrate and contact resistance at the active material/current collector interface) and the electrolyte resistance^72^.

We found relatively large values for the equivalent series resistance in both devices, which arise from the utilization of a gel electrolyte and, furthermore, a large part comes from the electrodes themselves, because we did not use a standard metallic current collector on the backside of silicon electrodes, taking the advantage of the low intrinsic resistance of the Si wafers (1–5 mΩ∙cm), even though their thickness was around 525 µm. In these circumstances, we obtained essentially good *R*_*s*_ of ~26 Ω for *NC_ J-SSC*, and even better, ~20 Ω, for *NC_CV-SSC*, demonstrating an improved interface formed between the active material and the bulk electrode. The values of the resistances faced by the electrolyte ions to access at different levels in porous structure, *R*_2_ and *R*_3_, respectively, clearly show that the barriers significantly increase for the lower level of hierarchy in the pore network. Accordingly, if *NC_ J-SSC* shows a good charge transfer resistance *R*_2_ (12.32 Ω), fivefold lower than the one of the *NC_CV* (59.21 Ω), attesting an enhanced electron transport, which facilitates fast redox reactions, the *R*_3_ is calculated to be ~348 Ω in both devices.

From the EIS plots the specific capacitance values (C_EIS_) were obtained at different frequencies (Fig. 5(c)), showing a similar relationship between the two supercapacitors like those attained from CV and GCD measurements. The complex capacitance analysis on the frequency reflects both the bulk and interfacial electrochemical properties. Thus, at low frequencies the SSCs reach full capacitance, because there is sufficient time for penetration of the electrolyte ions deep inside of the nanoporous electrode, promoting abundant faradaic reactions at interaction with the surface active sites. At higher frequencies, the electrolyte ions only access only the outer surface of the electrodes, and, in addition, the time interval becomes inadequate for slow faradaic charge storage, resulting a sharp decrease in capacitance^73^. The superior diversity of the surface functionalities involved in faradaic reactions that are present in *NC_ J-SSC* determines on one hand higher capacitance values (inset plots), and, on the other hand, gradual decrease of capacitance. Figure 5(d) shows the dependence of imaginary capacitances *vs*. frequency calculated from the relationship^74^ $\,C^{\prime} =\frac{{Z}_{re}}{\omega {|Z|}^{2}}$, which can be assigned to energy losses by irreversible processes. The relaxation time constants, describing the dielectric relaxation time characteristic of the complete system, were calculated using the values of the frequencies corresponding to the maximum capacitance (*τ* = *1/f*_*max*_), obtaining 1.93 s for *NC_CV-SSC* and 1 s for *NC_ J-SSC*, respectively. The calculated values are considerable superior than those reported for other symmetric supercapacitors like standard carbon/carbon (*τ* = 10 s)^75^, close to the value reported for hierarchical carbon nanosheet-based supercapacitors^76^. Thus, taking into account that *τ* represents essentially the minimum time required to deliver the stored energy effectively, the present values indicates a good ability of SSCs for rapid delivery of high power^75^.

The specific energy (*E*) and power (*P*) densities of the devices were calculated as following:

6 $$E=\frac{1}{2}\times \frac{C{({\rm{\Delta }}V)}^{2}}{3600}$$

7 $$P=\frac{E}{{\rm{\Delta }}t}\times 3600$$

where *C* is the specific capacitance of the prototype devices, *ΔV* is the voltage window and *Δt* is the discharge time.

Consequently, using the galvanostatic charging-discharging data at different current densities we obtained the Ragone plots corresponding to our new devices.

**Figure 6:**
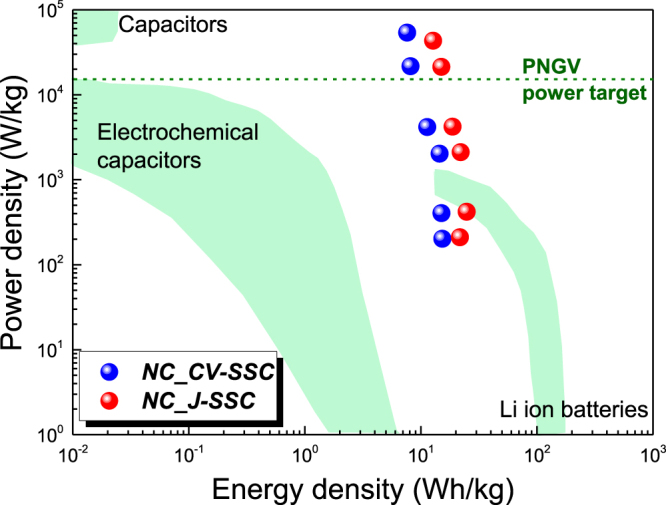
Ragone plots for *NC_CV-SSC* and *NC_ J-SSC* devices.

As Ragone plots show in Fig. 6, the power - energy curves of our new symmetric supercapacitors are situated in the superior right part of the graph, confirming the enhanced energy storage ability of our nano-carbon/porous silicon assemblies in relationship with the main electrical energy storage devices^77^. Accordingly, the maximum energy density of *NC_ J-SSC* (*NC_CV-SSC*) devices is about 25 Wh/kg (15 Wh/kg) at the power density of 420 W/kg (202 W/kg), while the maximum power density is about 54 kW/kg (43 kW/kg) at the energy density of 12.7 Wh/kg (7.6 Wh/kg) at a large operational potential window of 2.1 V. These values indicate that both supercapacitors exhibit on the one hand ultrafast ion and electron transport and, on the other hand handle high power figures (>30 kW/kg), which are superior than the power target of the Partnership of a New Generation of Vehicle (PNGV, 15 kW/kg)^78^. The obtained energy and power densities are highly competitive with those of other previously reported symmetric supercapacitor devices using porous silicon or carbon based materials. Detailed analysis regarding the specific capacitance and the operational voltage window are summarised in Table S3, in the Supporting Information section, in comparison with the energy and power characteristics of the best performing symmetric supercapacitors reported in the literature. Particularly concerning the nanocomposite carbon (graphene)/p-Si electrodes, although different methods have been used for their fabrication, the maximum energy density values are at least 1.5 times lower than our SSCs, ranging from 4.8 Wh/kg^24^ to 9 Wh/kg^79^ or 10 Wh/kg^26^. The corresponding power density has significantly lower maximum values in the first two cases, but it increases up to 65 kW/kg, 20% better than our results, when tetraethylammonium tetrafluoroborate dispersed in acetonitrile is used as ionic electrolyte^26^. The issue in this case is associated to the modest value of the corresponding energy density, only 1.3 Wh/kg, which is 6 times lower than the one achieved for *NC_CV-SSC* device. An increase in the energy density was reported only for asymmetric SCs based on nanocomposite carbon/silicon nanowires electrodes, when one of the electrodes was supplementary decorated with MnO_x_^34^. To the best of our knowledge, the highest reported performances for SSCs based on carbon materials are 95.7 Wh/kg in terms of energy density, achieved by a 2D quasi-ordered nitrogen-enriched porous carbon nanohybrids electrodes, but the delivered maximum power was only 29.7 kW/kg^80^, and ~110 kW/kg in terms of power density, achieved for a hierarchical microporous/mesoporous carbon nanosheets electrodes^76^. It is notable that in both cases the SSC devices were assembled using an organic electrolytes, Et_4_NBF_4_-PC and TEABF_4_/AN, respectively. Thus, even though remarkable values of energy and power densities were obtained for both devices, starting from the new proposed technology for fabrication of a graphene interconnected networks in porous silicon electrodes using electrochemical deposition techniques, the SSC performances can be further improved by tuning the electrode electrolyte interactions^67^.

**Figure 7:**
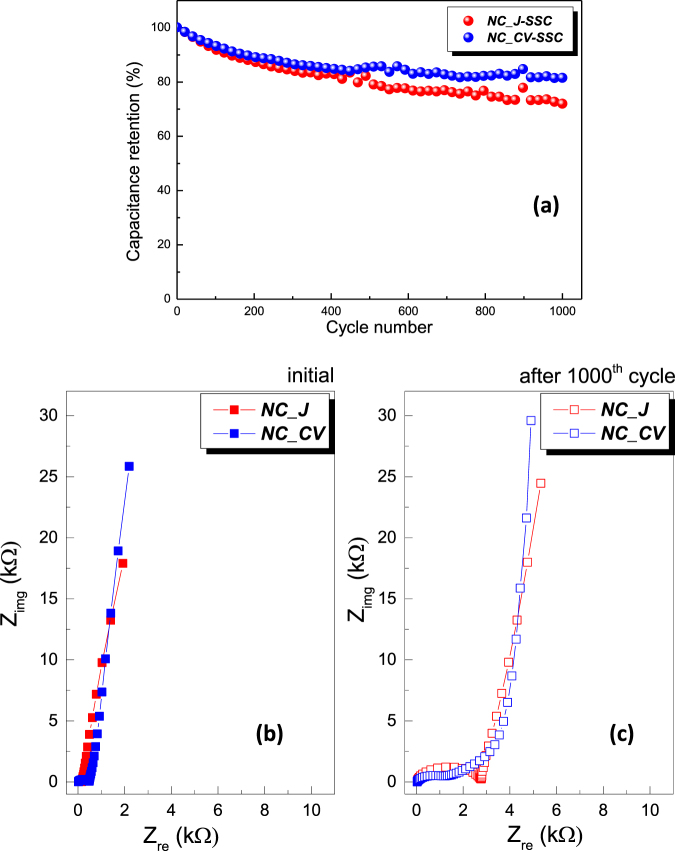
(**a**) Cycling stability at current density of 10 A/g. Comparative Nyquist plots recorded before (**b**) and after (**c**) 1000 cycles.

In addition, for practical applications, we measured the cycling stability and we analysed the alterations occurred in their electrochemical performances. When test cells were galvanostatically cycled at high current density of 10 A/g between −0.9 and 1.2 V for 1000 cycles, a relative good stability was obtained (Fig. 7(a)).

The hybrid electrodes demonstrate an improved stability in the case of *NC_CV-SSC*, keeping 83% of capacitance retention even at this high current density of 10 A/g. The superior performance of *NC_CV-SSC* may be ascribed to a better coverage of the porous silicon substrate with carbon as evidenced by the XPS survey, where C-O/C-OH chemical species and carbon relative concentration are higher on *NC_CV-SSC*. It is also notable that the cycling tests were performed at relatively high current density, where the pseudocapacitive contribution becomes limited. However, the cycling performance can be further improved through adjusting the concentration of electrolyte or electrolyte ions with different valence states. Analysing comparatively the EIS Nyquist diagrams obtained before and after cycling test (Fig. S6), a relatively different way of alteration can be observed. According to the fitting results presented in Table S2, the R_s_ values become almost equal, averaging around 28–29 Ω. However, if they were practically equal in the beginning, the R_3_ values get a dramatic increase for *NC_ J-SSC* that shows the obstruction of the charge access to the deeper level of the microstructure. This behaviour is confirmed also by the values obtained for the constant phase element related to Warburg diffusion, where a decrease of three orders of magnitude results for *NC_ J-SSC*. A different evolution is visible in the case of *NC_CV-SSC*, where it can be clearly observed that a wide-ranging, approximately linear segment emerges at intermediary frequencies, disposed at an angle to the real axis, which correspond to the migration of electrolyte deep inside of the porous structure leading to capacitive charging processes inside the pores with a wide distribution of time constants and finally arising a set of capacitances that charge and discharge asynchronously^81^. This behaviour may be related to cycling induced changes in pore structure, process amplified in the case of the faradaic electrodes, where, during charging/discharging cycling, especially at high currents, a microstructural agglomeration appears leading to their degradation, and reducing the electroactive sites^82^. The TEM characterization performed on the as-fabricated and cycled electrodes confirm this assumption and show that whereas the cycling process did not destroy or noticeably modify the quality of active *NC_CV* electrode, a physical degradation can be observed in the cycled *NC_ J* electrode, which apparently obstructs the channels for electrolyte ions access (Fig. S7). A supplementary confirmation was achieved analysing the voltammograms recorded before/after the 1000^th^ cycle, which are reasonably equivalent for *NC_CV-SSC*, whereas the disappearance of the main redox peaks ascribed to pseudocapacitive behaviour is clearly observed in the case of *NC_ J-SSC* (Fig. S8). Therefore, the densely deposited nanocarbon/interconnected graphene sheets in the *NC_CV* nanocomposite offer electric conductive paths within the microstructure, securing the cycling stability, and thus endorse high power densities. Furthermore, the existence of a higher internal surface area in *NC_CV* according to BET and SAXS measurements supports the improved stability, becoming available during the GCD cycling operation.

## Conclusions

In summary, we have successfully fabricated hybrid electrodes consisting of interconnected networks of graphene inside of the nanoporous silicon matrix using two electro-deposition processes, cyclic voltammetry or potentiometry, respectively, promoting synergistic interactions between silicon and the organic modifier and a beneficial combination of properties. When assembled as symmetric supercapacitors using a standard electrolyte (H_2_SO_4_-PVA), a comparative analysis of resulted *NC_CV-SSC* and *NC_ J-SSC* test devices’ performances for energy storage applications was completed. Both devices presented outstanding results at a large operational potential windows of 2.1 V, the hierarchical, hybrid pore structure with short diffusional paths, being able to handle ultra-high rates for voltage sweep, 5 V/s, or discharge current density, 100 A/g, respectively. Nonetheless, it is notable that whereas *NC_CV-SSC* distinguishes as ultra-high power delivering (53.8 kW/kg), the *NC_ J-SSC* exhibits high specific capacity of 162.4 F/g at 1 A/g and ultra-high energy density 24.8 Wh/kg, and these distinctions were determined by interplaying effects of a sum of individual characteristics of the electrodes: (i) compositional, the potentiometric electrodeposition determines an arrangement of the polymer backbones that promotes the faradaic reactions and adds a significant pseudocapacitive component to the overall capacitance; (ii) micro-structural, the polymerisation of 2,6-DHN using cyclic voltammetry leads to a thinner, more compact shell layer of carbon on silicon nano-walls that conserves the capacity storage during the stability tests. Overall, the new way of fabrication of valuable supercapacitor electrodes on silicon using electrochemical processes represents a simple and effective for the large-scale production of high-performance supercapacitors that might fill the gap between electrochemical capacitors and batteries.

## Electronic supplementary material

Supplementary file
