# Supplementary material for: High-performance solid state supercapacitors assembling graphene interconnected networks in porous silicon electrode by electrochemical methods using 2,6-dihydroxynaphthalen
Source: Sci Rep. 2018 Jun 25;8:9654. doi: 10.1038/s41598-018-28049-x (PMC6018509; doi:10.1038/s41598-018-28049-x)
Supplement: Supplementary file 1 — Supplementary file [file 41598_2018_28049_MOESM1_ESM.pdf]

# Supplementary Information

## High-performance solid state supercapacitors assembling graphene interconnected networks in porous silicon electrode by electrochemical methods using 2,6-dihydroxynaphthalen

Cosmin Romanitan[1,4], Pericle Varasteanu[1,4], Iuliana Mihalache[1], Daniela Culita[2], Simona Somacescu[2], Razvan Pascu[1], Eugenia Tanasa[1,3], Sandra A. V. Eremia[5], Adina Boldeiu[1], Monica Simion[1], Antonio Radoi[1*], Mihaela Kusko[1*]

[1] National Institute for Research and Development in Microtechnology (IMT-Bucharest), 126A Erou Iancu Nicolae Street, 077190, Voluntari, Romania

[2] 'Ilie Murgulescu' Institute of Physical Chemistry, Romanian Academy, 202, Splaiul Independentei, Bucharest 060021, Romania

[3] Faculty of Applied Sciences, Politehnica University of Bucharest, 313 Splaiul Independentei, Bucharest, 060042, Romania

[4] Faculty of Physics, University of Bucharest, 405 Atomistilor Street, 077125, Magurele, Romania

[5] National Institute of Research and Development for Biological Sciences, Centre of Bioanalysis, Bucharest, 296 Splaiul Independentei, Bucharest, 060031, Romania

**Corresponding authors:**

*E-mail: antonio.radoi@imt.ro; mihaela.kusko@imt.ro

**Figure S1.** X-ray reflectivity (XRR) measurements for ***NC_J*** and ***NC_CV*** electrode materials in comparison with ***p-Si substrate***.

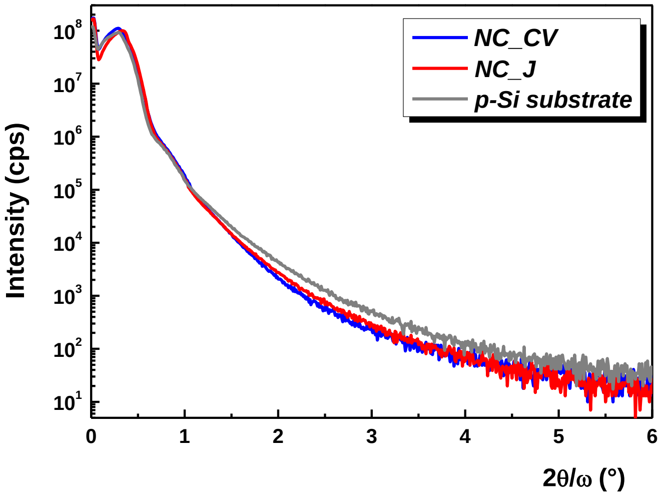


**Figure S2.** FTIR spectrum collected for ***p-Si substrate*** before the electrochemical deposition of 2,6-DHN.

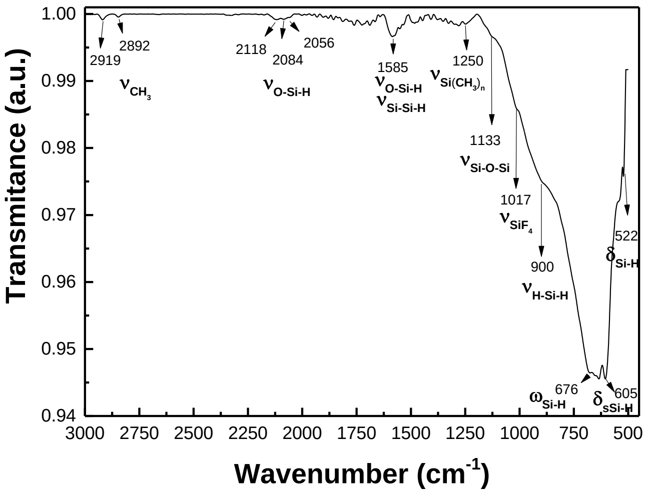


FTIR analysis of the freshly porosified silicon wafer revealed the presence of $CH_2$ groups assigned to the adsorbed molecules of ethanol and isopropanol used in the porosification procedure (see **Fabrication of the porous silicon (p-Si) and its modification** under **Experimental** section**)**. Residues of hydrofluoric acid were also revealed as $SiF_4$. The specific chemical groups for porous silicon are evidenced as O-Si-H, Si-O-Si, H-Si-H and Si-H [*Appl. Surf Sci.* **307**, 704-711 (2014)].

FTIR analysis was performed using a Vertex 80 v spectrometer from Brucker operating at 3 mbar, with a resolution of 2 $cm^{-1}$.

**Figure S3.** C1s high resolution deconvoluted photoelectron spectrum for the nanocomposite samples ***NC_J*** (a) and ***NC_CV*** (b), respectively.

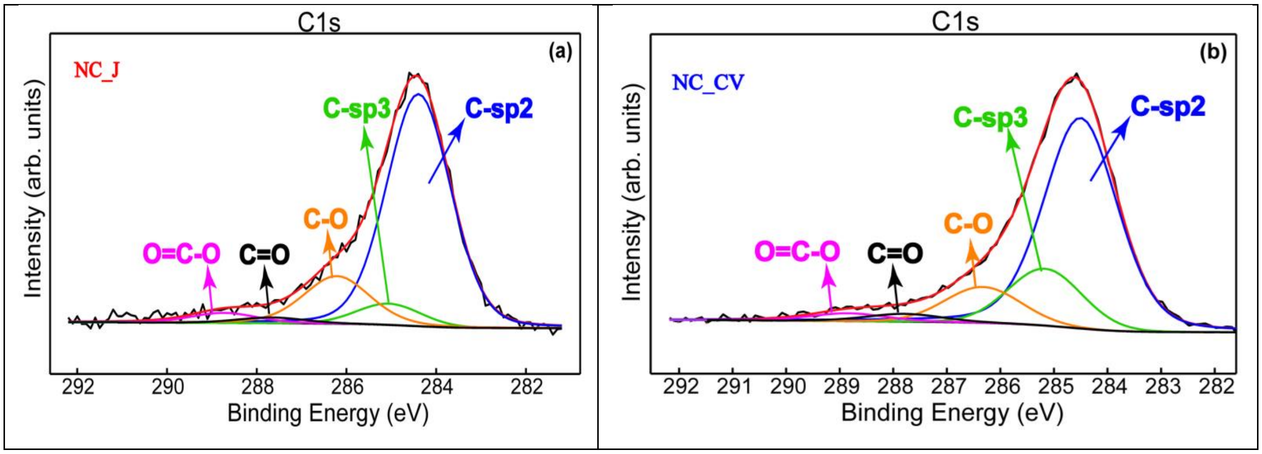


**Figure S4.** O1s (a) and Si2p (b) high resolution, superimposed photoelectron spectra for the samples ***NC_J*** and ***NC_CV***, respectively.

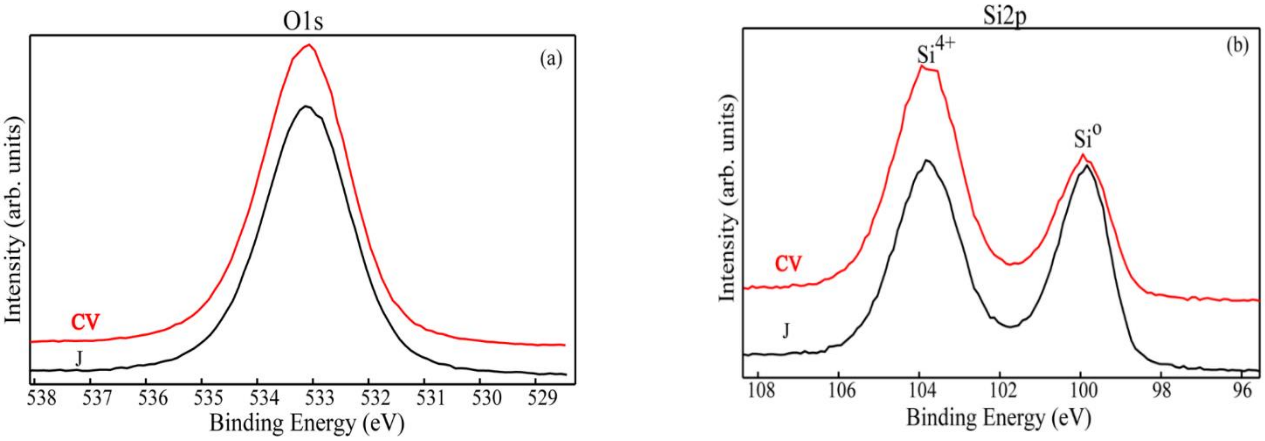

**Table S1**. Binding Energies (BEs, eV) and quantitative assessment: atomic relative concentrations (atom %) and chemical states relative concentrations (%).

| **Sample** | **Binding Energies (eV) / chemical state rel. conc. (%)** | | | **Atomic rel. conc. (atom %)** | | |
|---|---|---|---|---|---|---|
| | **C 1s** | **O 1s** | **Si 2p** | **C** | **O** | **Si** |
| **CV** | **C – sp$^2$**<br>284.5 eV (67.2%)<br>**C – sp$^3$**<br>285.1 eV (16.8%)<br>**C-O**<br>286.4 eV (10.9%)<br>**C=O**<br>287.8 eV (2.5%)<br>**O=C-O**<br>289.9 eV (2.6%) | 533.1 | **Si$^0$**<br>99.8 eV (~45.2%)<br>**Si$^{4+}$**<br>103.8 eV (~54.8%) | 29.2 % | 16.0 % | 54.8 % |
| **J** | **C – sp$^2$**<br>284.5 eV (72.4%)<br>**C – sp$^3$**<br>285.1 eV (6.3%)<br>**C-O**<br>286.4 eV (16.0%)<br>**C=O**<br>287.8 eV (1.9%)<br>**O=C-O**<br>289.9 eV (3.4%) | 533.2 | **Si$^0$**<br>99.8 eV (~34.2%)<br>**Si$^{4+}$**<br>103.8 eV (~65.8%) | 12.9 % | 21.3 % | 65.8 % |

**Figure S5.** $IR_{drop}$ at different charge/discharge current densities for ***NC_J-SSC*** (a) and ***NC_CV-SSC*** (b), respectively.

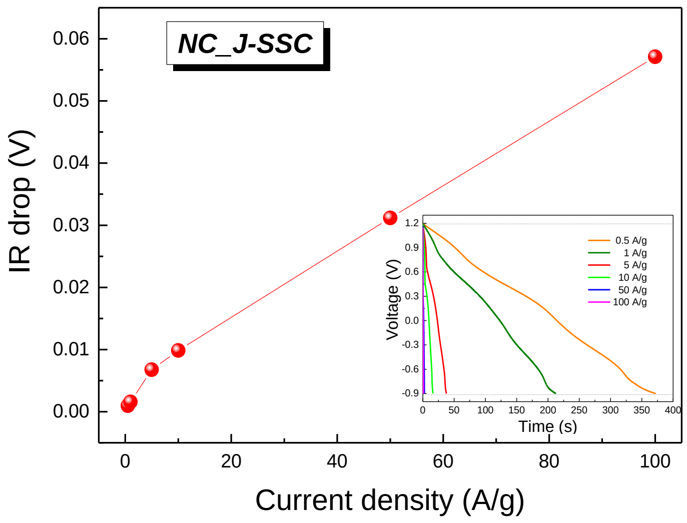


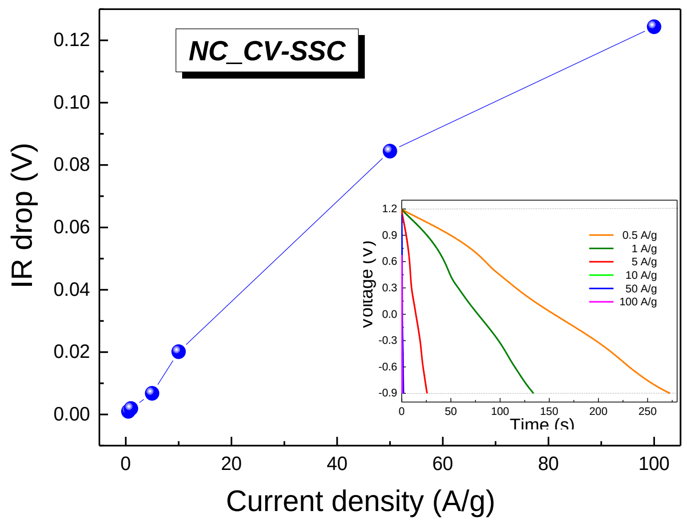

**Figure S6.** The data analysis of the EIS experiments - equivalent circuit model used for data fitting (a) and corresponding raw (points) and simulated (lines) data for Nyquist plots from our test devices: ***NC_J-SSC*** (b1; b2) and ***NC_CV-SSC*** (c1; c2).

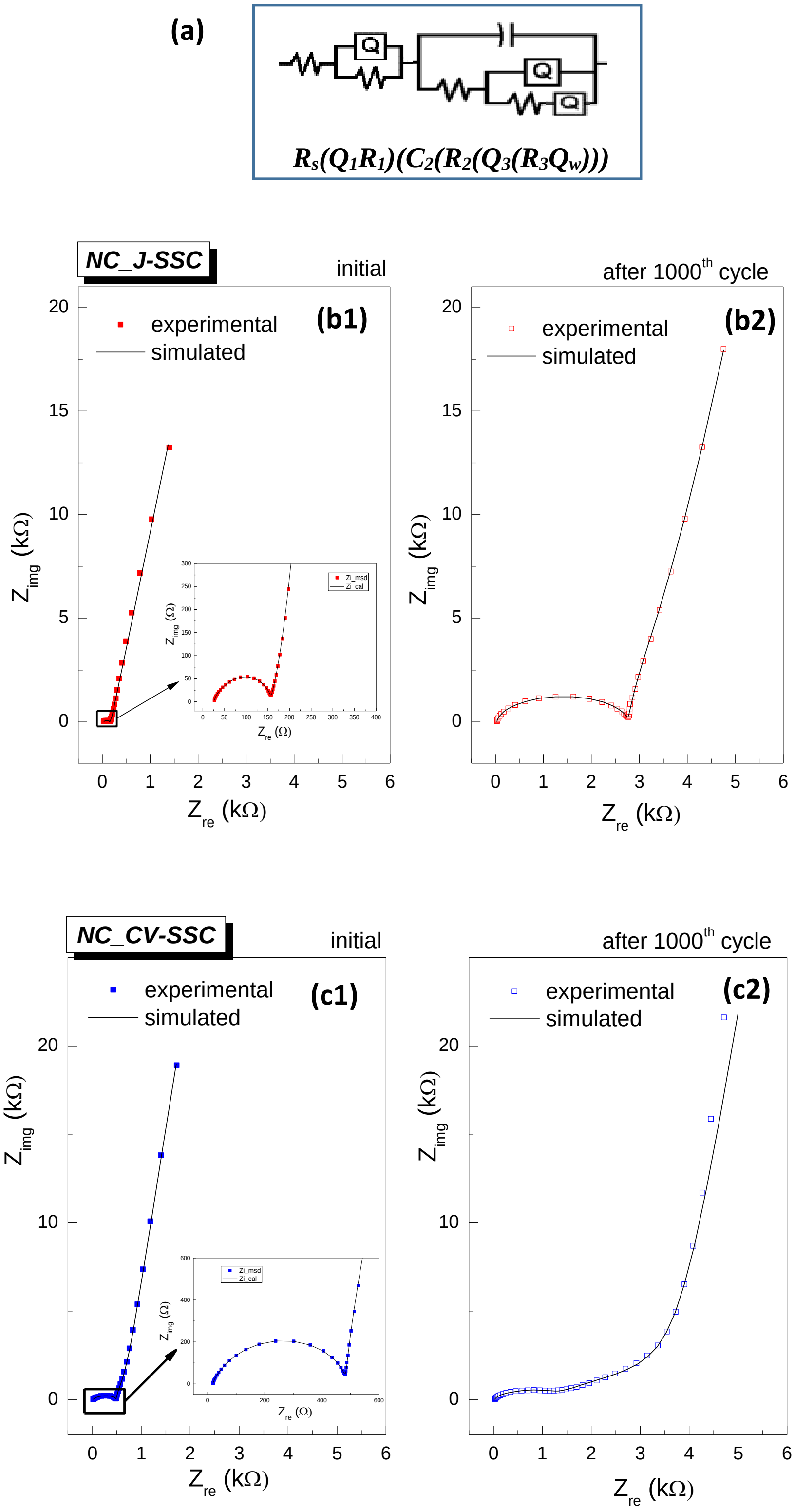

**Table S2.** The fitting parameters resulted from the equivalent circuit: $R_s(Q_1R_1)(C_2(R_2(Q_3(R_3Q_w)))$

| 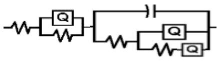 | **R** | **$Q_1$** | **$R_1$** | **$C_2$** | **$R_2$** | **$Q_3$** | **$R_3$** | **$Q_W$** | **$\chi^2$** |
|---|---|---|---|---|---|---|---|---|---|
| ***NC_J-SSC*** **– initial** | 25.64 | 2.58E-6<br>n=0.914<br>$C_1$ = 1.2 E-6 | 119.7 | 1.27E-6 | 12.32 | 0.55E-3<br>n=0.955<br>$C_3$ = 0.43E-3 | 348.6 | 0.19E-3<br>n=0.91<br>$C_W$ = 0.14E-3 | 5.3E-5 |
| ***NC_CV-SSC*** **– initial** | 20.22 | 5E-6<br>n=0.912<br>$C_1$ = 2.24E-6 | 49.42 | 0.8E-6 | 59.21 | 0,82E-6<br>n= 0,96<br>$C_3$ = 0.58E-6 | 348.1 | 0.525E-3<br>n=0,934<br>$C_W$ = 0.46E-3 | 3.7E-5 |
| ***NC_J-SSC*** **– after 1000$^{th}$ cycle** | 29.07 | 1.03E-6<br>n=0.943<br>$C_1$ = 0.72E-6 | 2620 | 4.45E-6 | 112.9 | 0.515E-3<br>n=0,95<br>$C_3$ = 0.63E-3 | 76.8E3 | 0.475E-6<br>n=0,903<br>$C_W$ = 0.55E-6 | 1.02e-4 |
| ***NC_CV-SSC*** **– after 1000$^{th}$ cycle** | 28.42 | 3.1E-6<br>n= 0,875<br>$C_1$ = 1.35E-6 | 964.9 | 0.16E-6 | 718.6 | 0,145E-3<br>n= 0,84<br>$C_3$ =0.13E-3 | 3982 | 0.27E-3<br>n= 0,97<br>$C_W$ = 0.27E-3 | 3.78e-4 |

**Figure S7.** Comparative TEM (a-d) and HR-TEM (e-f) images of ***NC_ J*** and ***NC_CV*** electrodes before (a, c, e) and after (b, d, f) cycling.

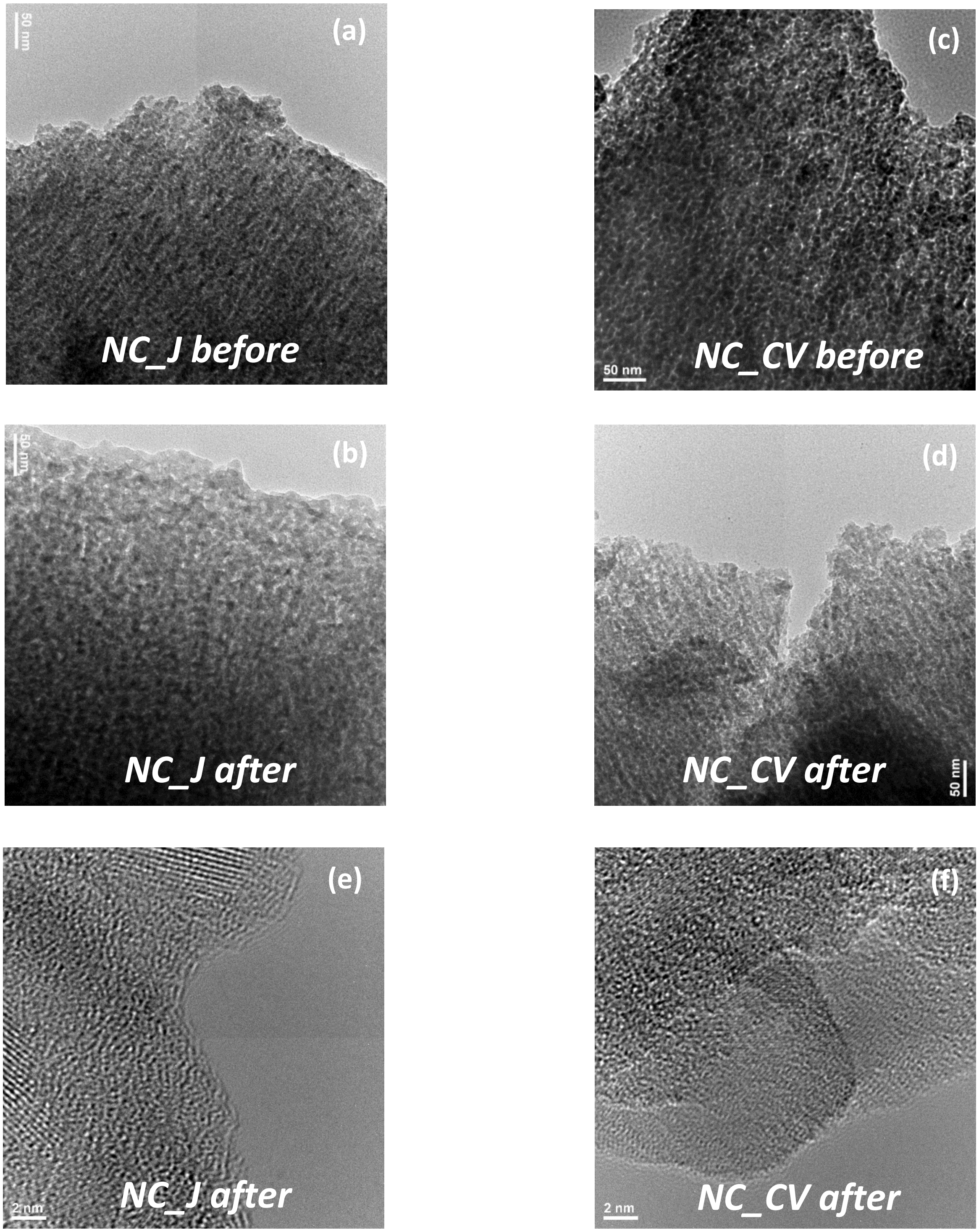

**Figure S8.** Comparative CV curves obtained at scan rates of 50 mV/s before and after 1000 GCD cycles for ***NC_J-SSC*** (a) and ***NC_CV-SSC*** (b) devices; (inset: artistic representation of polymer backbone).

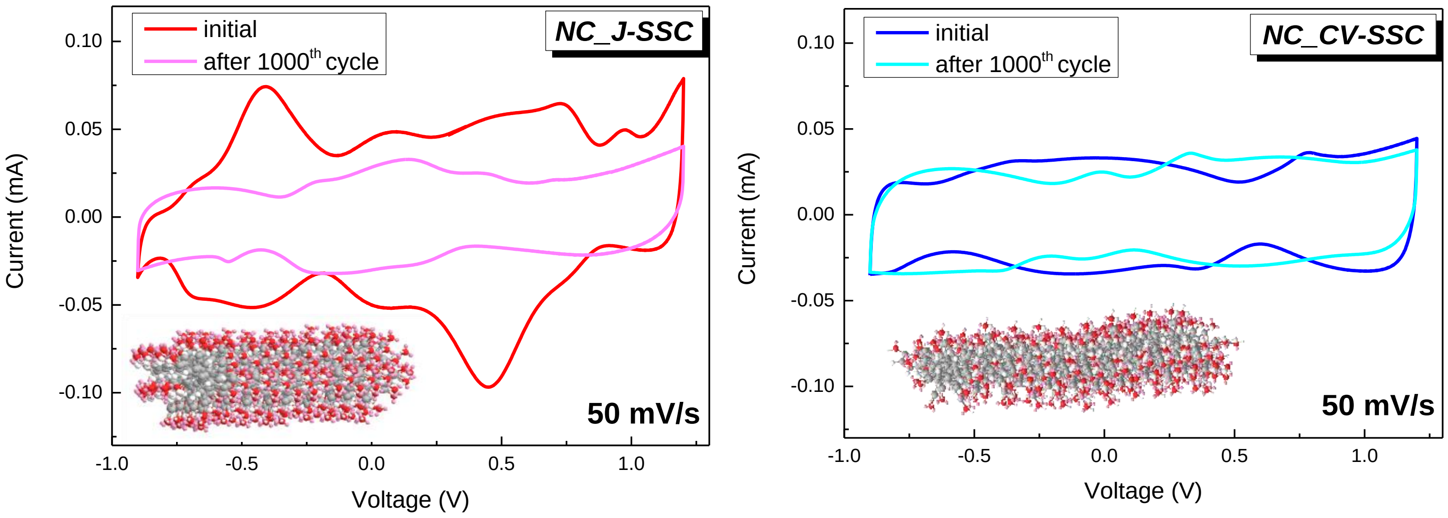

**Estimation of the active electrode mass**

The mass of active electrode materials are measured using a microbalance (XS 205 Mettler-Toledo, 0.01 mg precision) with 0.01 mg precision.

Thus, 4 Si wafers in different stages of fabrication (i.e. 1 original Si wafer (noted **Si**), 1 porosified Si wafer (noted **p-Si/Si**), 1 electrodeposited by potentiometric method and thermally treated (**NC-J/Si**) and 1 electrodeposited by cyclic voltammetry method and thermally treated (**NC-CV/Si**) were diced in rectangular 1.2x1.8 $cm^2$ chips using DAD322 Disco Automatic Dicing Saw machine and 10 of them were weighted to obtain an average mass, according to the **Figure S9**.

**Figure S9. NC-J/Si** samples used to estimate the ***NC_J*** active mass.

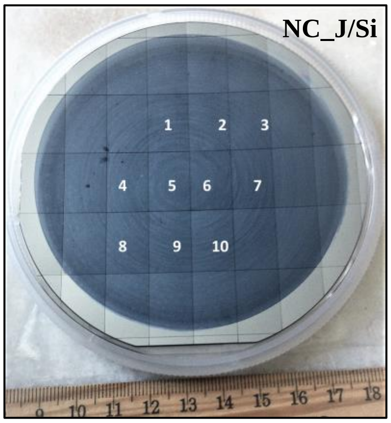


**Table S4.** Mass measurements of samples originating from **Si**, **p-Si/Si**, **NC-J/Si** and **NC-CV/Si** wafers, respectively.

| **Measurement** | **$m_{Si}$ (g)** | **$m_{p-Si/Si}$ (g)** | **$m_{NC-J/Si}$ (g)** | **$m_{NC-CV/Si}$ (g)** |
|---|---|---|---|---|
| S1 | 0,26437 | 0,26224 | 0,26261 | 0,26227 |
| S2 | 0,26439 | 0,26229 | 0,26248 | 0,26238 |
| S3 | 0,26439 | 0,26205 | 0,26233 | 0,26228 |
| S4 | 0,26444 | 0,26222 | 0,26260 | 0,26253 |
| S5 | 0,26463 | 0,26218 | 0,26221 | 0,26220 |
| S6 | 0,26474 | 0,26234 | 0,26252 | 0,26243 |
| S7 | 0,26456 | 0,26254 | 0,26263 | 0,26257 |
| S8 | 0,26457 | 0,26224 | 0,26231 | 0,26228 |
| S9 | 0,26463 | 0,26221 | 0,26223 | 0,26223 |
| S10 | 0,26433 | 0,26190 | 0,26192 | 0,26194 |
| **Average mass ($m_{av}$)** | **0,264505** | **0,262221** | **0,262384** | **0,262311** |
| Std. Dev. (σ) | 0,00013 | 0,00016 | 0,00022 | 0,00018 |

In estimating the active electrode mass, we included only the active porous region, subtracting the supporting Si substrate mass; the thickness of porous silicon layer is 5 µm and that of entire Si wafer is 525 µm. Accordingly, the following equations have been used to calculate the active mass of SSC electrodes:

- mass of porous silicon matrix before electro-polymerisation:

$$m_{p-Si} = m_{p-Si/Si} - \frac{520}{525} * m_{Si} \quad (1)$$

- mass of active nanocomposite electrodes:

$$m_{NC\_J} = m_{NC\_J/Si} - \frac{520}{525} * m_{Si} \quad (2)$$

$$m_{NC\_CV} = m_{NC\_CV/Si} - \frac{520}{525} * m_{Si} \quad (3)$$

- mass of graphene like material (***G_J*** and ***G_CV*** ) embedded in porous silicon matrix (neglecting the possible loss of Si during electrochemical deposition):

$$m_{G\_J} = m_{NC\_J} - m_{p-Si}$$

$$m_{G\_CV} = m_{NC\_CV} - m_{p-Si}$$

**Table S5.** Calculations of active mass of ***p-Si-SSC***, ***NC_J-SSC*** and ***NC_CV-SSC***, respectively, as well as mass of graphene like material (***G_J*** and ***G_CV***) embedded in porous silicon matrix for nanocomposite electrodes.

| | ***p-Si*** | ***NC_J*** | ***NC_CV*** | ***G_J*** | ***G_CV*** |
|---|---|---|---|---|---|
| **Mass (g)** | 2,37E-04 | 4,00E-04 | 3,27E-04 | 1,63E-04 | 9,00E-05 |
| **Samples' area (cm$^2$)** | 2,16 | 2,16 | 2.16 | 2,16 | 2,16 |
| **Mass per cm$^2$** | 1,10E-04 | 1,85E-04 | 1,51E-04 | 7,55E-05 | 4,17E-05 |
| **Mass corresponding to SSC active area (0.49 cm$^2$)** | 5,38E-05 | 9,08E-05 | 7,42E-05 | 3,70E-05 | 2,04E-05 |

**Table S3.** Summary of various SSCs figures of merit

| | Potential window | Specific capacitance | Energy density | Power density | REF |
|---|---|---|---|---|---|
| Pristine p-Si SSC, $EMIBF_4$ ionic electrolyte | 2.3 V | - | **$E_{max}$ = 0.3 Wh/kg** | P = 0.4 kW/kg | 1 |
| | | | E = 0.06 Wh/kg | **$P_{max}$ = 1.8 kW/kg** | |
| Graphene-coated p-Si, $EMIBF_4$ ionic electrolyte | 2.3 V | - | **$E_{max}$ = 4.8 Wh/kg** | P = 0.8 kW/kg | |
| | | | E = 2.5 Wh/kg | **$P_{max}$ = 3.5 kW/kg** | |
| Ultrathin, graphene-like carbon coatings performed directly on the p−Si by CVD using PEO:$EMIBF_4$ ionic electrolyte | 3 V | - | **$E_{max}$ = 9 Wh/kg** | P = ~1 kW/kg | 2 |
| | | | E = 1.4Wh/kg | **$P_{max}$ = 8 kW/kg** | |
| Graphene-passivated porous silicon SSC using TEATFB ionic electrolyte | 2.6 V | - | **$E_{max}$= 10 Wh/kg** | P= 1 kW/kg | 3 |
| | | | E = 1.3 Wh/kg | **$P_{max}$ = 65 kW/kg** | |
| $MnO_x$/C/PSiNWs//C/PSiNWs *asymmetric* SSC, 0.1M EMIM-TFSI ionic liquid electrolyte. | 3.6 V | - | **$E_{max}$ = 244 Wh/kg** | P = 213 W/kg | 4 |
| | | | E = 34 Wh/kg | **$P_{max}$ = 24 kW/kg** | |
| Carbon nanofibers - bridged porous carbon nanosheets, 1 M $Na_2SO_4$ electrolyte | 1.8 V | - | **$E_{max}$ = 20.4 Wh/kg** | P = 81.8 W/kg | 5 |
| | | | E = 7.4 Wh/kg | **$P_{max}$ = 17.8 kW/kg** | |
| Sandwiched porous carbon layer/graphene hybrids, 1 M $Na_2SO_4$ electrolyte | 1.8 V | 481 F /g at 0.5 A/g<br>313 F/g at 20 A/g | **$E_{max}$ = 25.7 Wh/kg** | P = 100 W/kg | 6 |
| | | | E = 20 Wh/kg | **$P_{max}$ = 40 kW/kg** | |
| Immense surface area carbons (ISACs), 1M $Li_2SO_4$ electrolyte | 1.8 V | 177 F/g at 15 A/g | **$E_{max}$ = 29 Wh/kg** | P = 442 W/kg | 7 |
| | | | E = 17 Wh/kg | **$P_{max}$ = 3.94 kW/kg** | |
| Carbon materials with hierarchical porosity, 6M KOH electrolyte | 1 V | 250 F /g at 0.5 A/g<br>140 F/g at 100 A/g | **$E_{max}$ = 28.3 Wh/kg** | P = 150.2 W/kg | 8 |
| | | | E = 17.2 Wh/kg | **$P_{max}$ = 3 kW/kg** | |
| Hierarchical carbon nanosheets, 1M $H_2SO_4$ electrolyte | 1 V | 140 F/g at 150 A/g | **$E_{max}$ = 8 Wh/ kg** | P = 100 W/kg | 9 |
| | | | E = 2 Wh/kg | **$P_{max}$ = 30 kW/kg** | |
| Hierarchical carbon nanosheets, $TEABF_4$/AN electrolyte | 2.7 V | ~95 F/g at 150 A/g | **$E_{max}$ = 30 Wh/ kg** | P = 70 W/kg | |
| | | | E = 16 Wh/kg | **$P_{max}$ = ~110 kW/kg** | |
| Activated carbon electrodes PVA-$H_2SO_4$-HQ electrolyte | 1 V | 500 F/g at 0.5 A/g | **$E_{max}$ = 18.7 Wh/ kg** | P = 245 W/kg | 10 |
| | | | E = 4 Wh/kg | **$P_{max}$ = 3.5 kW/kg** | |
| Polyaniline based carbon nanospheres with PVA/$H_2SO_4$/AQSA-Na gel electrolyte | 1.5 V | 430 F/g at 0.8 A/g | **$E_{max}$ = 33.4 Wh/kg** | P = 600 W/kg | 11 |
| | | | E = 25 Wh/kg | **$P_{max}$ = 2.25 kW/kg** | |
| Polyaniline based carbon nanospheres, PVA/$H_2SO_4$ gel electrolyte | 1.5 V | 230 F/g at 0.8 A/g | **$E_{max}$ = 19 Wh/kg** | P = 600 W/kg | |
| | | | E = 15 Wh/kg | **$P_{max}$ = 2.25 kW/kg** | |

| | | | | | |
|---|---|---|---|---|---|
| 2D quasi-ordered nitrogen-enriched porous carbon nanohybrids, 1 M $Et_4NBF_4$-PC ionic electrolyte | 3 V | 72.7 F/g at 1 A/g<br>~55 F/g at 20 A/g | **$E_{max}$ = 95.7 Wh/kg** | P = 449.7 W/kg | 12 |
| | | | E = 69.5 Wh/kg | **$P_{max}$ = 29.7 kW/kg** | |
| N-doped carbon nanosheets/VN nanoparticles as positive electrode, $Ni(OH)_2$ negative electrode, KOH electrolyte | 1.6 V | 89.6 F/g at 0.5 A/g<br>13 F/g at 20 A/g | **$E_{max}$ = 29.5 Wh/kg** | P = 385 W/kg | 13 |
| | | | E = 4.28 Wh/g | **$P_{max}$ = 15.4 kW/kg** | |
| ***NC_J-SSC*** | 2.1 V | 142 F/g at 0.5 A/g<br>83 F/g at 100 A/g | **$E_{max}$ = 24.8 Wh/kg** | P = 420 W/kg | ***This work*** |
| | | | E = 12.7 Wh/kg | **$P_{max}$ = 43.2 kW/kg** | |
| ***NC_CV-SSC*** | 2.1 V | 100 F/g at 0.5 A/g<br>50 F/g at 100 A/g | **$E_{max}$ = 15.3 Wh/kg** | P = 202 W/kg | |
| | | | E = 7.6 Wh/kg | **$P_{max}$ = 53.8 kW/kg** | |

## References


[1] Oakes L., Westover A., Mares J. W., Chatterjee S., Erwin W. R., Bardhan R., Weiss S. M. & Pint C. L. Surface engineered porous silicon for stable, high performance electrochemical supercapacitors, *Scientific Reports* **3**, 3020 (2013).

[2] Westover A. S., Tian J. W., Bernath S., Oakes L., Edwards R., Shabab F. N., Chatterjee S., Anilkumar A. V. & Pint C. L. A multifunctional load-bearing solid-state supercapacitor. *Nano Lett.* **14**, 3197–3202 (2014).

[3] Chatterjee S., Carter R., Oakes L., Erwin W. R., Bardhan R. & Pint C. L. Electrochemical and corrosion stability of nanostructured silicon by graphene coatings: toward high power porous silicon supercapacitors. *J. Phys. Chem. C* **118**, 10893- 10902 (2014).

[4] Ortaboy S., Alper J. P., Rossi F., Bertoni G., Salviati G., Carraro C. & Maboudian R. $MnO_x$-decorated carbonized porous silicon nanowire electrodes for high performance supercapacitors. *Energy Environ. Sci.* **10**, 1505-1516 (2017).

[5] Presser V., Zhang L., Niu J. J., McDonough J., Perez C., Fong H. & Gogotsi Y. Flexible nano-felts of carbide-derived carbon with ultra-high power handling capability. *Adv. Energy Mater.* **1**, 423–430 (2011).

[6] Yan J., Wang Q., Lin C., Wei T. & Fan Z. Interconnected frameworks with a sandwiched porous carbon layer/graphene hybrids for supercapacitors with high gravimetric and volumetric performances. *Adv. Energy Mater.* **4**, 1400500 (2014).

[7] Pokrzywinski J., Keum J. K. Ruther R. E. Self E. C., Chi M., Meyer III H., Littrell K. C., Aulakh D., Marble S., Ding J., Wriedt M., Nanda J. & Mitlin D. Unrivaled combination of surface area and pore volume in micelle-templated carbon for supercapacitor energy storage. *J. Mater. Chem. A* **5**, 13511-13525 (2017).

[8] Liu X., Liu X., Sun B., Zhou H., Fu A., Wang Y., Guo Y.-G., Guo P., Li H. Carbon materials with hierarchical porosity: Effect of template removal strategy and study on their electrochemical properties. *Carbon* **130**, 680-691 (2018).

[9] Fuertes A. B. & Sevilla M. Hierarchical microporous/mesoporous carbon nanosheets for high-performance supercapacitors. *ACS Appl. Mater. Interfaces* **7**, 4344–4353 (2015).

[10] Zhong J., Fan L.-Q., Wu X., Wu J.-H., Liu G.-J., Lin J.-M., Huang M.-L. & Wei Y.-L. Improved energy density of quasi-solid-state supercapacitors using sandwich-type redox-active gel polymer electrolytes. *Electrochim. Acta* **166** 150-156 (2015).

[11] Feng E., Peng H., Zhang Z., Li J. & Lei Z. Polyaniline-based carbon nanospheres and redox mediator doped robust gel films lead to high performance foldable solid-state supercapacitors. *New J. Chem.* **41**, 9024-9032 (2017).

[12] Kan K., Wang L., Yu P., Jiang B., Shi K.& Fu H. 2D quasi-ordered nitrogen-enriched porous carbon nanohybrids for high energy density supercapacitors. *Nanoscale* **8**, 10166-10176 (2016).

[13] Tan Y., Liu Y., Tang Z., Wang Z., Kong L., Kang L., Liu Z. & Ran F. Concise N-doped carbon nanosheets/vanadium nitride nanoparticles materials via intercalative polymerization for supercapacitors. *Scientific Reports* **8**, 2915 (2018).
